# Innovative assessment of lipid‐induced oxidative stress and inflammation in harvested human endothelial cells

**DOI:** 10.14814/phy2.16048

**Published:** 2024-06-14

**Authors:** Mohammad Saleem, Taseer Ahmad, Alexandria Porcia Haynes, Claude F. Albritton, Naome Mwesigwa, Meghan K. Graber, Annet Kirabo, Cyndya A. Shibao

**Affiliations:** ^1^ Department of Medicine, Division of Clinical Pharmacology, Room 536 Robinson Research Building Vanderbilt University Medical Center Nashville Tennessee USA; ^2^ Department of Pharmacology, College of Pharmacy University of Sargodha, University Road Sargodha Punjab Pakistan; ^3^ School of Graduate Studies, Meharry Medical College Nashville Tennessee USA; ^4^ Vanderbilt Center for Immunobiology, Vanderbilt University Medical Center Nashville Tennessee USA; ^5^ Vanderbilt Institute for Infection, Immunology and Inflammation Nashville Tennessee USA; ^6^ Vanderbilt Institute for Global Health Nashville Tennessee USA

**Keywords:** flow cytometry, inflammation, J‐wire technique; endothelial cells, lipid, oxidative stress

## Abstract

Studying acute changes in vascular endothelial cells in humans is challenging. We studied ten African American women and used the J‐wire technique to isolate vein endothelial cells before and after a four‐hour lipid and heparin infusion. Dynamic changes in lipid‐induced oxidative stress and inflammatory markers were measured with fluorescence‐activated cell sorting. We used the surface markers CD31 and CD144 to identify human endothelial cells. Peripheral blood mononuclear cells isolated from blood were used as a negative control. The participants received galantamine (16 mg/day) for 3 months. We previously demonstrated that galantamine treatment effectively suppresses lipid‐induced oxidative stress and inflammation. In this study, we infused lipids to evaluate its potential to increase the activation of endothelial cells, as assessed by the levels of CD54+ endothelial cells and expression of Growth arrest‐specific 6 compared to the baseline sample. Further, we aimed to investigate whether lipid infusion led to increased expression of the oxidative stress markers IsoLGs and nitrotyrosine in endothelial cells. This approach will expedite the in vivo identification of novel pathways linked with endothelial cell dysfunction induced by oxidative stress and inflammatory cytokines. This study describes an innovative method to harvest and study human endothelial cells and demonstrates the dynamic changes in oxidative stress and inflammatory markers release induced by lipid infusion.

## 1 INTRODUCTION

The endothelium plays a key regulatory role in vascular homeostasis (Colvert et al., 2023; Deanfield et al., 2007; Oates et al., 2022). An imbalance in nitric oxide (NO) and reactive oxygen species (ROS) or oxidative stress promotes endothelial dysfunction, leading to cardiovascular disease, including hypertension and myocardial infarction (Gudjoncik et al., 2014; Higashi et al., 2014). Endothelial dysfunction is the initial step in the pathogenesis of arteriosclerosis, resulting in cardiovascular complications (Goligorsky, 2005; Ross, 1999). Oxidative stress and pro‐inflammatory processes are often deemed mutually dependent, with many studies suggesting that oxidative stress is a direct stimulus to inflammation and vice versa (Kim et al., 2006; Saleem et al., 2016, 2018). Our previous work found that nicotinamide adenine dinucleotide phosphate (NADPH) oxidase activation and ROS production contribute to inflammation and cardiovascular disease by forming highly immunogenic isolevuglandins (IsoLGs)‐protein adducts (Förstermann et al., 2017; Wu et al., 2016).

Growth arrest‐specific 6 (GAS6) promotes endothelial cell activation and facilitates interactions between endothelial cells, platelets, and leukocytes (Tjwa et al., 2008). GAS6 is a protein‐coding gene, and elevated levels of plasma GAS6 are found in numerous pathological conditions, including sepsis, obesity, chronic renal disease, cardiac hypertrophy, and systemic lupus erythematosus (Ekman et al., 2011; Lee et al., 2012; Wu et al., 2015; Zhao et al., 2016). Also, it activates the TAM (Tyro‐3, Axl, and Mer) tyrosine kinase receptor family and is involved in the stimulation of cell proliferation (Fernandez‐Fernandez et al., 2008). Recently, we identified the activated endothelium as the source of GAS6 and this was associated with increased IsoLG‐adduct formation (Van Beusecum et al., 2021). Additionally, GAS6 contributes to monocyte transformation into a proinflammatory, IL‐1β‐producing phenotype (Tjwa et al., 2008; Van Beusecum et al., 2021). These data suggest that GAS6 represents a biomarker for endothelial dysfunction and a potential therapeutic target for modulating inflammation.

Previous work has shown that African American individuals have a greater oxidative stress response to lipid infusion at doses of 0.8 mL/m^2^/min with heparin (200 U/hrs for 4 hrs) than in Whites (Lopes et al., 2003), which is confirmed by plasma levels of the oxidative stress biomarker F2‐isoprostane (Parsa et al., 2022; Saleem et al., 2023). However, a reliable protocol to measure dynamic changes in oxidative stress responses in human endothelial cells has yet to be developed. The prior methodologies were used to isolate human endothelial cells, but contamination and poor yields were major concerns. We thus sought to develop a minimally invasive technique to obtain endothelial cells from human subjects with higher yields and purity. This protocol represents a novel method to collect and identify endothelial cells and the dynamic changes in oxidative stress and inflammatory markers before and after lipid infusion.

## 2 METHOD

### 2.1 Ethical statement and volunteer characteristics

The study was conducted at the Vanderbilt Clinical Research Center (CRC). The volunteers gave written informed consent before enrolling in the study as approved by the Institutional Review Board (IRB) of Vanderbilt University. All procedures were performed according to the Declaration of Helsinki. The African American women volunteers (BMI >28) were included in the study, while the exclusion criteria include individuals with a history of physician‐diagnosed myocardial infarction, angina, heart failure, stroke, uncontrolled hypertension defined as persistent blood pressure >140/90, diabetes mellitus type 1 or type 2, as defined by fasting plasma glucose of 126 mg/dL or greater hemoglobin A1C (HbA1C) 6.5%, the use of nitrates, pregnancy or breastfeeding and women, seizures or history of seizures and current smokers. Table 1 describes the demographic and clinical characteristics of the volunteers who participated in the study. Data is available upon request from the corresponding author.

**TABLE 1:** Demographic details of participants.

Study ID	Age	Sex	Race	BMI	Blood pressure (supine)	Heart rate
023–55,509	25.6	Female	African American	28.9	134/74	80
024–55,509	27.3	Female	African American	50	132/80	79
025–55,509	36.7	Female	African American	40	141/81	57
026–55,509	27.3	Female	African American	45.4	112/82	77
027–55,509	45.3	Male	African American	40	145/90	59
028–55,509	20.5	Female	African American	33.1	120/65	76
029–55,509	38.1	Male	African American	29	107/68	42
030–55,509	35.4	Female	African American	29.6	138/86	66
031–55,509	35	Female	African American	29.3	123/81	66
032–55,509	56.4	Male	African American	45.5	134/81	63

## 3 RESULTS

**FIGURE 1:**
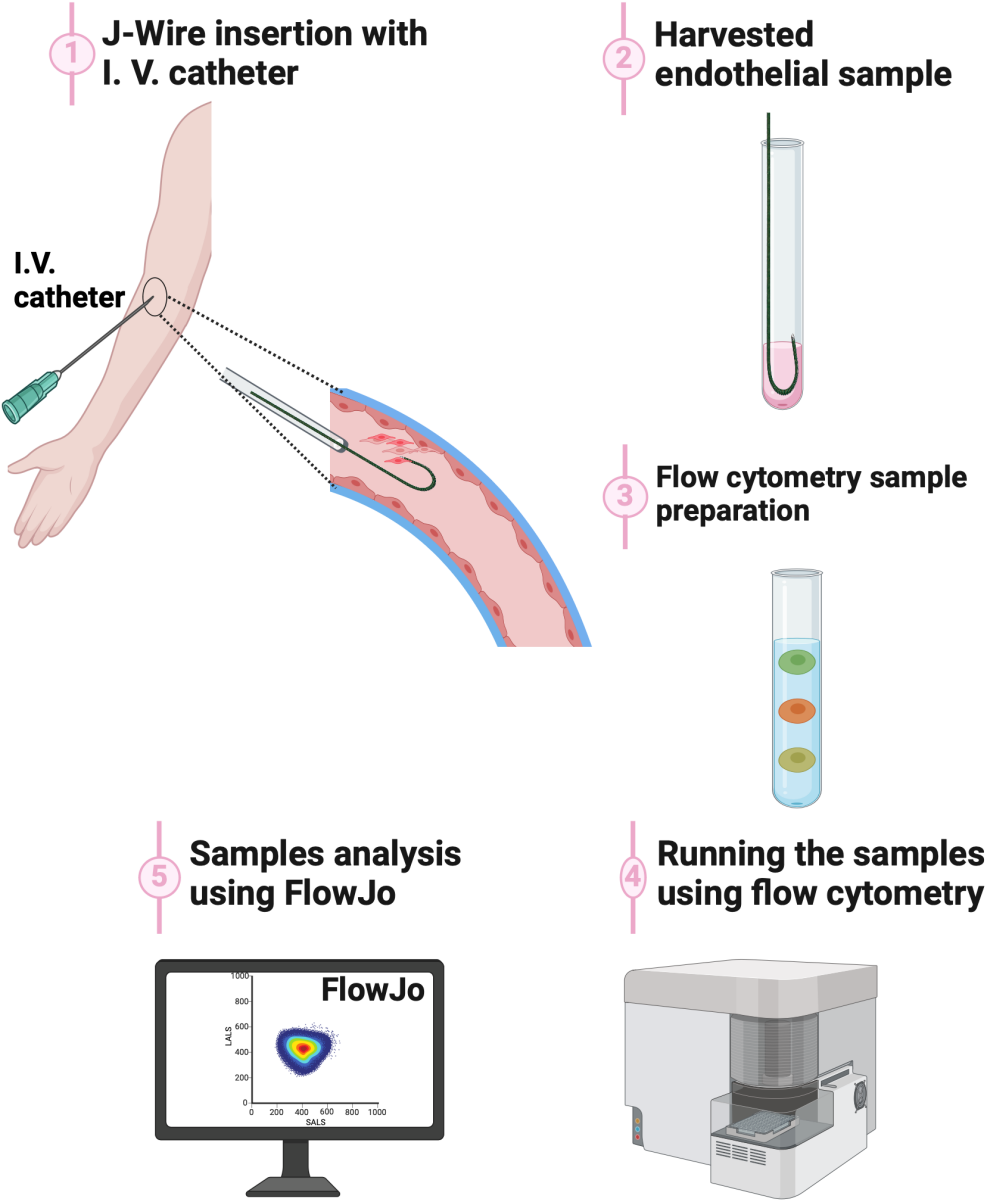
Pictorial depiction of J‐Wire minimally invasive method to sample human endothelial cells. The human endothelial cells were harvested from the intimal surface of the cubital vein using J‐wire, and analysis was performed with flow cytometry (Image was created using BioRender).

**FIGURE 2:**
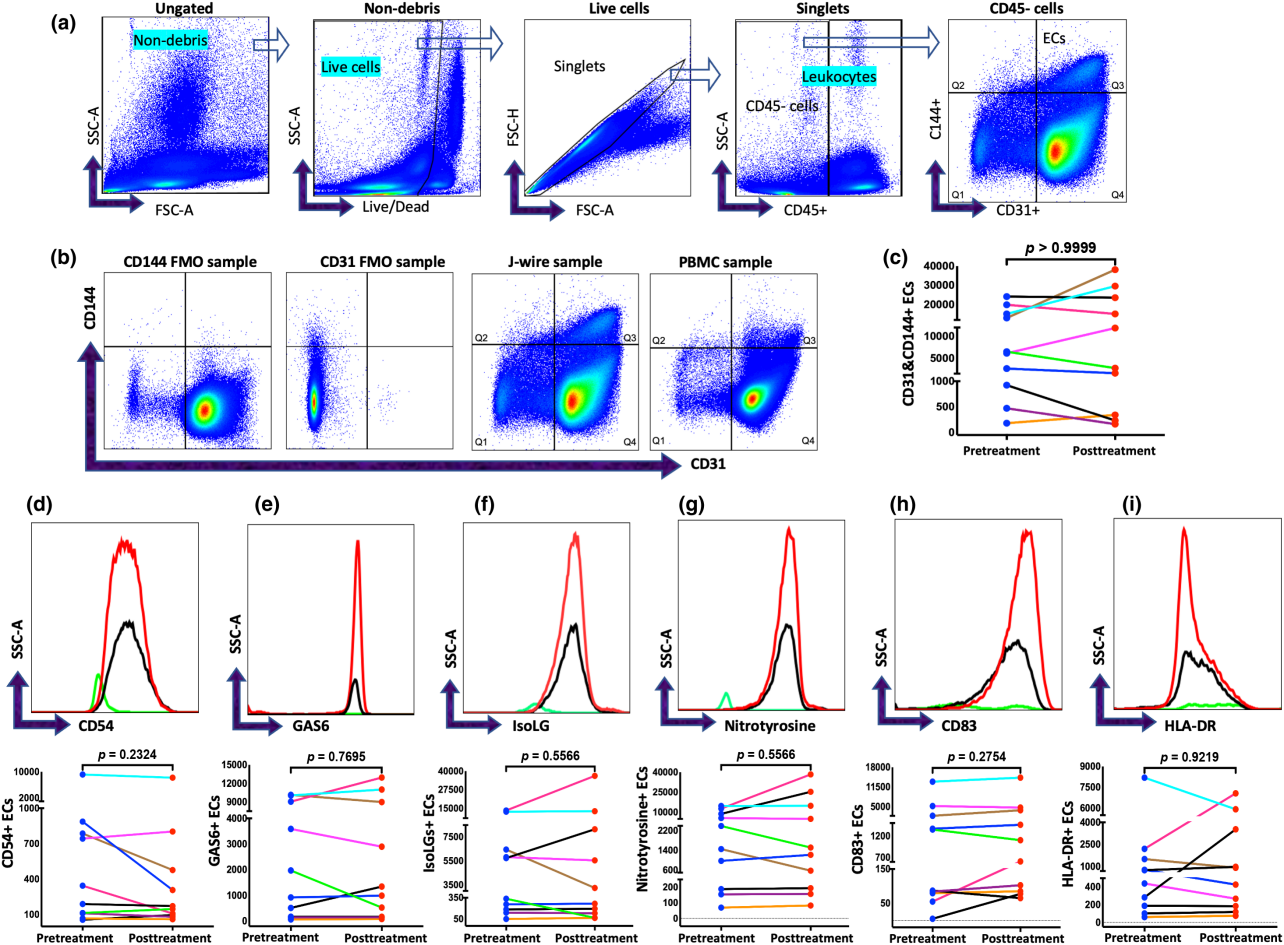
Flow cytometric analysis of endothelial cells (ECs) and peripheral blood mononuclear cells (PBMCs) isolated from volunteers. (a) Gating strategy for identifying endothelial cells. Cells were gated on SSC‐A versus FSC‐A to exclude debris and subsequently gated for live cells. Live cells were sub‐gated to identify singlets. Within this population, cells were gated on SSC‐A versus CD45+ to identify CD45‐negative and CD45‐positive populations. From the CD45‐negative population, we gated endothelial cells using CD31 and CD144 endothelial‐specific markers. (b) Showing the specificity of the antibody used to detect endothelial cells and the PBMC sample used as a negative control. (c) Quantification of endothelial cells (ECs). (a) A histogram showing the expression of CD54, (b) GAS6, (c) IsoLG, and (d) nitrotyrosine on endothelial cells isolated with the J‐wire technique before (pretreatment) and after lipid infusion (Posttreatment). The Green color indicates the FMO sample, black pretreatment, and red indicates the posttreatment sample. We selected an experiment that shows the activation of endothelial cells and increased levels of oxidative stress after lipid infusion as a representative sample. Under the histograms, bar graphs show the quantification of their respective molecules before and after the treatment. In bar graphs, each color represents an individual participant. For the comparison, we employed the Wilcoxon signed rank test to conduct paired analysis.

To determine if we isolated the endothelial cells with the J‐Wire method and if lipid infusion increased the activation of endothelial cells and the expression of oxidative stress markers in endothelial cells, we gated the cells, as shown in Figure 2a. The double‐positive CD144 (VE‐cadherin) and CD31 (PECAM1) cells were identified as endothelial cells (Figure 2b, Q3 quadrant in the J‐Wire sample). CD144 is a distinct marker expressed by endothelial cells, whereas CD31 is expressed by endothelial cells and some leukocytes and platelets. CD54, or ICAM‐1, is a protein expressed on activated endothelial cells, so CD54 was used in our analysis to identify activated endothelial cells. Figure 2c shows the number of endothelial cells isolated before and after lipid infusion for each participant. Further, to assess the dynamic changes in the levels of oxidative stress in endothelial cells before and after lipid infusion, we assessed endothelial activation markers CD54 and GAS6 and the expression of oxidative stress and inflammatory markers IsoLGs and nitrotyrosine from cells collected before and after lipid infusion treatment (Figure 2d–i). Posttreatment cells showed increased expression of endothelial cell activation markers CD54 and GAS6, as well as oxidative stress markers IsoLGs and nitrotyrosine in the participant, used for the representative histograms (Figure 2d–i). A detailed description of the protocol and procedure performed for the method is given below and depicted in Figure 1. Since we are still blinded to the treatment, we could not determine the outcome of the lipid infusion in the patients.

### 3.1 Protocol

#### 3.1.1 Use of lipid and heparin infusion to stimulate oxidative stress and inflammation

Following our established protocol, we infused 20% lipids (Intralipids, Baxter Healthcare Crop. Glendale, CA) at 0.8 mL/m^2^/min for 4 h (Parsa et al., 2022). An initial heparin bolus of 1000 U was administered, followed by a 200 U/h infusion during the 4‐hour period of lipid infusion to activate endothelial lipoprotein lipase on the vascular endothelium, which hydrolyzes nonesterified free fatty acids (NEFA) from triglycerides during lipid infusion.

#### 3.1.2 Harvesting the endothelial cells using J‐Wires

Ten volunteers underwent peripheral blood and endothelial cell harvesting from the median cubital vein, as depicted in Figure 1. First, an 18‐gauge peripheral intravenous catheter was placed in the antecubital vein under semisterile conditions using a standard technique. We then inserted the 35 cm J‐wires (Catalog# AW‐04025‐J, Teleflex Medical, Ireland) sequentially into the previously placed peripheral intravenous cannula, and the endothelial cells were harvested using four sterile J‐wires from the intimal surface of the median cubital vein. The wire was gently advanced into the blood vessel (~5 cm beyond the tip of the catheter) and retracted through an 18‐gauge intravenous catheter. The retractions were performed 10 times, while external compression was applied to the vein to increase contact between the wire and vessel wall. The endothelial cells were harvested from the contralateral arms before and after lipid infusion to reduce the risk of vascular damage and oxidative stress that retractions may cause.

After the harvest, the wires were transferred to a dissociation buffer solution (phosphate‐buffered saline [PBS], 2 mM ethylenediamine tetra‐acetic acid [EDTA], heparin 0.1 mg/mL, pH 7.4) and were washed 10 times with PBS to detach endothelial cells. After centrifugation (350 × g for 7 min at room temperature with no brake), we aspirated dissociation buffer and resuspended cells in MACS buffer, and harvested endothelial cells were used for flow cytometric analysis.

#### 3.1.3 Endothelial cell phenotyping with flow cytometry

We performed flow cytometry for the endothelial cells to determine if we harvested the endothelial cells and what the dynamic changes in the activation and oxidative stress markers are before and after lipid infusion. First, we performed live–dead staining, and for that, we used Zombie NIR to exclude dead cells (1–2 μg Zombie NIR/100 μL MACS buffer). Other surface staining markers include the cluster of differentiation (CD) 31, CD54, CD144, CD45, CD14, CD16, HLA‐DR, and CD83 (also shown in Table 1). We used a 1:100 ratio for all antibodies unless otherwise mentioned (1 μL antibody/100 μL MACS buffer). The CD31 and CD144 double‐positive cells were determined as endothelial cells (Figure 2b, Q3 quadrant in the J‐wire sample). We use reagent A to fix the cells (Life Technologies, Cat # GAS001S100) for 20 min in the dark at room temperature.

To assess the intracellular markers, intracellular staining was performed for IsoLG (an adduct of lipid peroxide and D11 protein), nitrotyrosine, and GAS6. For the permeabilization, reagent B was used (Life Technologies, Cat # GAS002S100) to prepare the intracellular antibody with a 1:100 ratio. We attached a pacific orange fluorophore to the GAS6 antibody using a commercially available kit (Thermofisher, Cat# P30014). Similarly, Alexa fluor 488 fluorophore was attached to the D11 antibody using a commercially available kit (Thermofisher, Cat# A10468). We used a 1:25 ratio for these two antibodies conjugated in the lab.

We used fluorescence minus one to adjust gates and identify the correct population of endothelial cells, oxidative stress, and inflammatory markers (Figure 2b. CD144 FMO and CD31 FMO). The antibodies and other reagents used for the study are listed in Table 2. Data analysis was performed using the FlowJo application (Tree Star, Inc.).

**TABLE 2:** Antibodies and other materials used in this study.

Marker antihuman	Fluorochrome	Clone	Catalog number	Company
Antibodies used for surface staining				
Live dead	Zombie NIR	N/A	423,106	Biolegend
CD31	PE‐CY7	WM59	303,118	Biolegend
CD54	PerCP‐Cy5.5	HA58	353,120	Biolegend
CD45	BV750	2D1	368,542	Biolegend
CD62L (HLA‐DR)	Brilliant Violet 605 (BV605)	L243	307,640	Biolegend
CD83	APC/Cy7	HB15e	305,330	Biolegend
Antibodies used for intracellular staining				
CD11 (IsoLG)	Alexa Flour 488	N/A	901,509	Thermofisher
Antinitrotyrosine	Cy3		bs‐8551R	Bioss antibodies
GAS 6	Pacific Orange	D3A3G	67202S	Cell signaling technology

Other reagents				
Item	Description		Catalog number	Company
Intralipid	It comprises of 20% soybean oil, 1.2% egg yolk phospholipids, 2.25% glycerin, and water for injection. In addition, sodium hydroxide has been added to adjust the pH so that the final product pH is 8. The pH range is 6 to 8.9.			Baxter Healthcare Crop.
Heparin				
J‐Wire	The wires were used to retract the cells from the vessels		AW‐04025‐J	Teleflex Medical, Ireland
Dissociation buffer solution	The wire is kept in this buffer after endothelial cells harvest and before being used for flow cytometry			

## 4 DISCUSSION

The endothelium plays a key regulatory role in vascular homeostasis (Colvert et al., 2023; Deanfield et al., 2007; Oates et al., 2022). An imbalance in NO and ROS or oxidative stress promotes endothelial dysfunction, leading to cardiovascular disease, including hypertension and myocardial infarction (Gudjoncik et al., 2014; Higashi et al., 2014). Endothelial dysfunction is the initial step in the pathogenesis of arteriosclerosis, resulting in cardiovascular complications (Goligorsky, 2005; Ross, 1999). Studying human endothelial cells to assess the dynamic changes in vivo is difficult. The literature suggests that the circulating endothelial cells (CEC) number is very minute and documented that normal adults have 2.6 ± 1.6 CEC per milliliter of peripheral blood (Lin et al., 2000; Solovey et al., 1997). To overcome these issues, we used the J‐Wire technique and combined it with flow cytometry to isolate and study the dynamic changes in the vascular endothelial cells ex vivo. Continuous lipid infusion with intralipids, known to be safe in humans, was used to stimulate oxidative stress in participants. Lipids contain linoleic and oleic acids, which can activate protein kinase C and stimulate the production of ROS (Förstermann et al., 2017). We and others found increased F2‐IsoPs in African American volunteers compared to white subjects using this approach (Lopes et al., 2003). The high efficiency and yield of endothelial cells from the blood vessels are the main advantages of this method. When coupled with the flow cytometry method, the most important advantage of the J‐wire method is that we were able to see the dynamic changes in the oxidative stress markers of endothelial cells in the same patient. Compared to other methods, this is a minimally invasive and cost‐effective method that requires relatively simple equipment and reagents. The isolated endothelial cells can be used for various downstream assays, including cell culture, immunostaining, gene expression analysis, and functional studies. The disadvantages include the high degree of technical precision and skill to perform this method and the limited or restricted accessibility of blood vessels in certain patient populations or clinical settings. Although comparatively minimally invasive, it still requires accessing blood vessels, which carries inherent bleeding risks, infection, or damage to the surrounding structure. As with any tissue isolation technique, there is a risk of contamination during the J‐wire procedure, which can compromise the purity and integrity of the isolated endothelial cells. The varying number of isolated endothelial cells in each draw is another disadvantage that may affect the statistical power and generalizability of research findings.

Two specific surface staining markers (CD31, CD144) were used to identify endothelial cells, and intracellular markers were used to measure oxidative stress and inflammation. CD45 is universally expressed in leukocytes. Thus, this marker was used in our gating strategy to distinguish between leukocytes (CD45+) and nonleukocytes (CD45‐). CD31, also known as platelet endothelial cell adhesion molecule 1 (PECAM‐1), is a glycoprotein expressed on endothelial cells and leukocytes. Cadherin‐5, or VE‐cadherin (vascular endothelial cadherin), also known as CD144, is a distinct marker for endothelial cells (Flores‐Nascimento et al., 2015). After excluding leukocytes (CD45+ cells), CD31 and CD144 double‐positive cells were identified as endothelial cells (Caligiuri, 2020; Flores‐Nascimento et al., 2015). CD54, or ICAM‐1, is a protein expressed on activated endothelial cells (Hubbard & Rothlein, 2000). CD54 was used in our analysis to identify activated endothelial cells (Lawson & Wolf, 2009).

Intracellular straining markers D‐11, nitrotyrosine, and GAS6 were used to assess oxidative stress in endothelial cells. A single‐chain antibody D‐11 is used to detect IsoLG‐protein adducts (Kirabo et al., 2014). Inflammation has been implicated in the pathogenesis of hypertension, and recent evidence suggests that isolevuglandins (IsoLGs)‐protein adducts play a role. Several hypertensive stimuli contribute to forming IsoLG‐protein adducts, including excess dietary salt and catecholamines. ROS oxidizes arachidonic acid, leading to the formation of IsoLGs, which noncovalently adduct to lysine residues and alter protein structure and function (Xiao et al., 2018). In oxidative stress, nitrotyrosine is formed due to the nitration of protein‐bound and free tyrosine residues by reactive peroxynitrite molecules (Bandookwala & Sengupta, 2020). GAS6 amplifies endothelial cells' activation in response to inflammatory stimuli (Tjwa et al., 2008). The differences in the results endpoints are insignificant because we are still blinded to the treatment of these participants. Considering that the patients also received galantamine, which diminishes NADPH oxidase activity and lowers oxidative stress levels (Parsa et al., 2022), galantamine may have mitigated the inflammatory and oxidative impact of lipid infusion in the patients.

In sum, through our rigorous methodology utilizing the J‐wire technique, we have consistently achieved reproducibility in isolating endothelial cells. Here, we show that we successfully isolated endothelial cells using the J‐Wire method, as validated by performing flow cytometry. This approach will expedite the identification of novel pathways linked with endothelial cell dysfunction induced by oxidative stress and inflammatory cytokines.

## FUNDING INFORMATION

NIH, National Heart, Lung, and Blood Institute (NHLBI): Annet Kirabo R01 HL157584, R01HL144941, R03HL155041; Cyndya A. Shibao R01 HL159203, 5UL1TR002243–03; American Heart Association (AHA): Cyndya A. Shibao 967,054; NIH, National Center for Advancing Translational Sciences (NCATS). Cyndya A. Shibao 5UL1TR002243–03; R03HL155041. Mohammad Saleem AHA 23CDA1053072.

## CONFLICT OF INTEREST STATEMENT

All the authors have disclosed their funding support, CAS is currently a consultant for Antag Therapeutics, Theravance Biopharma and Argenx. The others authors do not have any conflict of interest to declare.

## ETHICS STATEMENT

The study was approved by the Vanderbilt Human Research Protection Program. All subjects provided written consent. We present original data.

## Supporting information


Supplementary File 1.
