# Supplementary figures and images for: Innovative assessment of lipid‐induced oxidative stress and inflammation in harvested human endothelial cells
Source: Physiol Rep. 2024 Jun 14;12(11):e16048. doi: 10.14814/phy2.16048 (PMC11176576; doi:10.14814/phy2.16048)

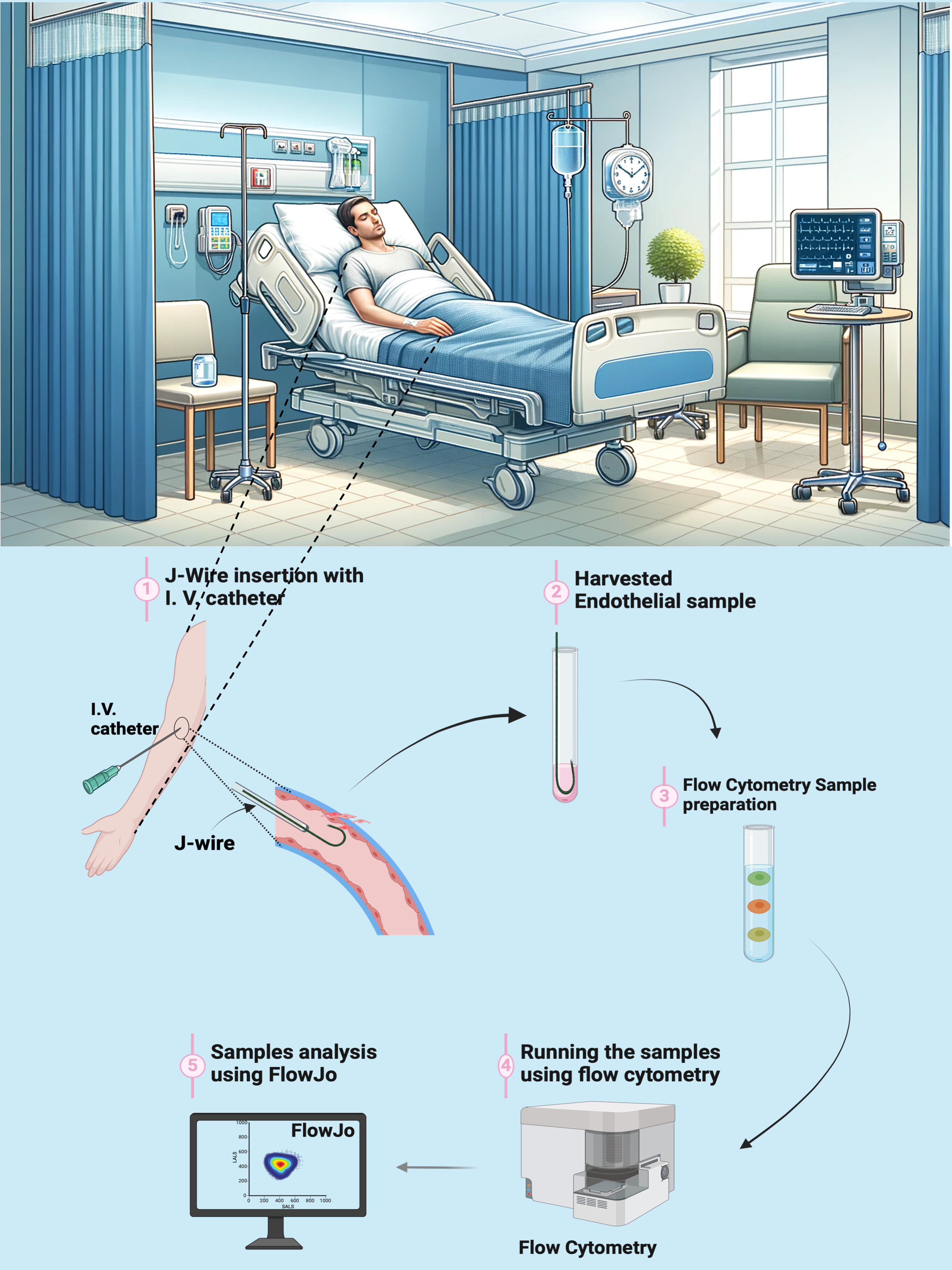

Supplement: Supplementary file 1 [file PHY2-12-e16048-s001.tif]
